# Corncob waste derived carbon with sulfonic acid group: An efficient heterogeneous catalyst for production of ethyl levulinate as biodiesel additives

**DOI:** 10.1016/j.heliyon.2024.e37687

**Published:** 2024-09-10

**Authors:** Dian Ratna Suminar, Cinantya Zahrina Pribadi, Qonita Rahmah Fitriana, Eko Andrijanto, Muhamad Diki Permana, Diana Rakhmawaty Eddy, Iman Rahayu

**Affiliations:** a Department of Chemistry, Faculty Mathematics and Natural Sciences, Universitas Padjadjaran, Sumedang, 45363, Indonesia; b Department of Chemical Engineering, Politeknik Negeri Bandung, Bandung Barat, 40559, Indonesia; c Special Educational Program for Green Energy Conversion Science and Technology, Integrated Graduate School of Medicine, Engineering, and Agricultural Sciences, University of Yamanashi, Kofu, 4008511, Japan; d Center for Crystal Science and Technology, University of Yamanashi, Kofu, 400-8511, Japan

**Keywords:** Levulinic acid, Esterification, Ethyl levulinate, Sulfonated carbon, Corncob

## Abstract

Alkyl levulinate is a biomass-based chemical compound used as a fuel additive. This research aims to produce ethyl levulinate from levulinic acid and ethanol using esterification with the assistance of a heterogeneous sulfonated carbon catalyst. The carbon sulfonate catalyst is obtained from corncob waste that has undergone carbonization at 300 °C and sulfonation using sulfuric acid at a temperature of 150 °C for 8 h. The catalyst is used for esterification with predetermined operating variables using Box-Behnken Design (BBD) on the response surface methodology (RSM). The result shows significant variables for ethyl levulinate esterification are catalyst loading and esterification time. The FTIR analysis indicates the presence of S=O bonds in the sulfonated carbon catalyst. The XRD and SEM analysis shows that the sulfonated carbon catalyst is in amorphous and mesoporous form. Catalyst reusability demonstrates that the corncob-derived carbon sulfonate catalyst can be used up to 3 times. The optimum condition for esterification is 9 h of reaction, 10 % catalyst loading, and a molar ratio of levulinic acid to ethanol of 1:10, which has 83.15 % conversion. These results present the optimum parameter conditions for an efficient heterogeneous catalyst from corncob for producing ethyl levulinate.

## 1 Introduction

Fossil energy is depleting as the world's population increases and global energy consumption rises [1]. Continuous use of fossil energy cannot meet future energy needs [2]. The use of fossil energy contributes to global warming, which is an environmental concern [3]. Renewable energy sources like biomass can provide an alternative solution to the energy problem [4]. Lignocellulosic waste from biomass poses a challenge in many countries, necessitating the utilization of biomass waste to create more useful products [5]. Biomass is a specific type of renewable new energy source that can produce carbon-based fuels or specialized chemicals for a clean and sustainable future [6].

Biomass, especially lignocellulosic biomass (LB), is one of the carbon-based resources that can be used to produce biofuels and high-value chemicals due to its abundant availability, low cost, biodegradability, low toxicity, and environmental friendliness [[7], [8], [9]]. One of the biomass wastes in Indonesia is corncob waste. Corncob waste contains cellulose (41 %), hemicellulose (36 %), lignin (6 %), and other common plant compounds. This indicates that corncob have the potential as a carbon source [10]. For example, the conversion of biomass into biofuels or vegetable fuels includes biodiesel. Biodiesel is often referred to as “green fuel” or environmentally friendly fuel because it is biodegradable, a renewable energy source, non-toxic, and safe for the environment [11]. The quality of biodiesel can be improved by adding additives to reduce toxicity, enhance lubrication, and improve the cold flow properties, thus enhancing biodiesel performance at low temperatures [12].

Alkyl levulinate can be used as a biodiesel additive. The use of alkyl levulinate includes being an additive in fuels, an essential substance in agriculture and pharmaceuticals, a food additive, a solvent, a polymer, and in chemical industries [13]. Alkyl levulinate in the forms of methyl, ethyl, and n-butyl levulinate can be produced through the esterification process of levulinic acid with alcohol using an acid catalyst [14,15].

In the production of alkyl levulinate, a catalyst is required. The catalyst has a high surface area and contains acid sites that play a crucial role in the esterification catalysis of levulinic acid [13]. Catalysts can be categorized into homogeneous and heterogeneous catalysts [16]. Homogeneous catalysts have several disadvantages such as leading to corrosion and toxic, causing environmental pollution, including their solubility in the products, making separation challenging [17]. Heterogeneous catalysts, on the other hand, have the advantage of being easily separable from the products, reusable, and more efficient [18].

Previous research has been conducted on the production of alkyl levulinate using various catalysts. A sulfonated bagasse-carbonized solid acid catalyst was used for the esterification of levulinic acid. The reaction was carried out at 120 °C for 9 h with an ethanol to levulinic acid molar ratio of 5:1, resulting in a yield of 88.2 mol% [19]. Carbon cryogel catalyst was employed in the esterification of levulinic acid at a temperature of 120 °C for 4 h with an ethanol to levulinic acid ratio of 15:1 and a catalyst loading of 25 wt%, resulting in a conversion of 87.2 % [20]. In other research, an amino sulfonated functional carbon material (NS-FCM) catalyst was used in the synthesis of ethyl levulinate at 120 °C for 8 h with an ethanol-to-levulinic acid ratio of 8:1, and a catalyst loading of 5 wt%, resulting in a conversion of 72 % [21].

Sulfonated carbon has been prepared from various natural sources such as loofah sponges and corn stalks [22,23]. The results showed satisfactory levulinic acid conversion of more than 90 % over 8–10 h. Natural sources contain a lot of carbon which is generally used in chemical processes because of its high surface area and porosity, making it suitable for use as a heterogeneous catalyst [24]. This carbon-based catalyst is prepared via sulfonation, which involves introducing H_2_SO_4_ into carbon pores to produce strong −SO_3_H groups [25,26]. Carbon-based acid catalysts are well documented as effective catalysts in the production of alkyl levulinates because they exhibit thermal stability, water tolerance, high acid sites, and good catalytic activity [27].

In this research, a heterogeneous acid catalyst was produced from corncob waste. Sulfuric acid is used as an activator of sulfonic groups. The use of carbon from corncob can be used to support catalyst production. The resulting sulfonated carbon catalyst was applied in the synthesis of ethyl levulinate. In this research, the response surface methodology (RSM) was used to determine the optimum conditions for three parameters, namely esterification time, catalyst loading, and molar ratio of reactants.

## 2 Materials and methods

### 2.1 Materials

The main raw material for making catalysts is corncob waste (*Zea mays* L.) obtained from local shops in Bandung, Indonesia. For activation and sulfonation of carbon, sulfuric acid (H_2_SO_4_, 96 %, Merck) was used. In addition, to test the acid concentration and catalyst activity, sodium hydroxide (NaOH, 99 %, Merck), hydrochloric acid (HCl, 37 %, Merck), phenolphthalein (PP, C_20_H_14_O_4_, Merck), ethanol (C_2_H_5_OH, 96 %, Merck), and levulinic acid (CH_3_C(O)CH_2_CH_2_CO_2_H, 98 %, Merck) were used.

### 2.2 Synthesis of sulfonated carbon catalyst

The pretreatment process for corncob waste includes cleaning the waste thoroughly using running water. Next, the corncob were crushed using a mortal and dried at 105 °C for 2 h. Then, the dried samples were carbonized at 300 °C. In this process, corncob will turn into charcoal. After carbonization, the charcoal was finely ground and sieved using a 125 μm mesh. After that, the carbon is functionalized to become sulfonate. Carbon was reacted with concentrated H_2_SO_4_ at a temperature of 150 °C for 8 h, with a carbon-to-H_2_SO_4_ ratio of 1:10 in a two-neck flask. After the sulfonation process is complete, the catalyst is washed with distilled water until the solution after washing becomes neutral. Then the catalyst is dried at a temperature of 105 °C until a constant weight is reached.

### 2.3 Characterizations

The catalysts were characterized using X-ray diffraction (XRD, Rigaku/MiniFlex 600, Tokyo, Japan) to identify the samples. The measurements were carried out at room temperature using Cu Kα radiation (λ = 0.15418 nm). To identify the morphology of the samples, scanning electron microscopy with electron dispersive spectroscopy (SEM-EDS, TM3030 Plus, Hitachi, Tokyo, Japan) was performed with a voltage of 15.0 kV. The samples were further characterized using Attenuated Total Reflection Fourier-transform infrared spectroscopy (ATR-FTIR, Jasco FT/IR-4700, Tokyo, Japan) to determine the functional groups in the composites. The surface areas of the samples were measured based on a multipoint Brunauer–Emmett–Teller (BET) method, pores size and pore volume were determined based on Barrett-Joyner-Halanda (BJH) method by nitrogen adsorption-desorption isotherm measurements at 77 K, using a BELSORP-mini analyzer (BEL JAPAN Inc, Tokyo, Japan). A thermogravimetry differential thermal analysis (TG-DTA) curve of the samples was obtained on a thermos plus (TG-DTA8120, Rigaku, Tokyo, Japan) in atmosphere gas condition with a heating rate of 10 °C/min.

### 2.4 Determination of moisture content and acid concentration

Moisture content is determined by weighing corncob, then the corncob are ground and dried at 105 °C for 2 h. Then, the volumetric method was used to determine the acid concentration in the catalyst. A total of 0.1 g of catalyst was added to 30 mL of 0.1 mol L^−1^ NaOH solution at room temperature for 1 h, followed by centrifugation. The filtrate obtained was then titrated using 0.1 mol L^−1^ HCl solution and phenolphthalein (PP) indicator. This step is done in triplicate. The acid concentration (mmol g^−1^) calculation is as follows Eq. (1), where *n*B is moles of NaOH (mmol), *n*A is moles of HCl (mmol), and w is the weight of the catalyst (g) [28].

(1) $$\text{Acid}\;\text{concentration}=\frac{n_{B}\;-\;n_{A}}{w}$$

### 2.5 Catalyst activity test using response surface methodology (RSM)

The determination of operating variables was performed to predict the optimum value using the Box−Behnken Design (BBD) for the response surface methodology (RSM). BBD with three factors and three levels was used in this study (Table 1). The experiments were conducted using the Minitab 17 software. The reactant mole ratio of levulinic acid and ethanol (*x*_1_), catalysts loading (*x*_2_), and esterification time (*x*_3_) were used as the independent variables, and levulinic acid conversion was recorded as the experimental response (*y*).

**Table 1:** Independent variables and their levels in the Box–Behnken Design.

Factor/Independent Variables	Low (−1)	Medium (0)	High (+1)
*x*_1_ = reactant mole ratio (levulinic acid:ethanol)	1:10	1:15	1:20
*x*_2_ = catalysts loading (%)	5	10	15
*x*_3_ = esterification time (h)	5	7	9

The esterification process was carried out using reflux equipment and a three-necked flask placed in a water bath, with variations in reactant mole ratio. Sulfonated carbon catalysts were slowly added to the three-necked flask while stirring at 250 rpm. The esterification process was conducted at 65 °C with variations in time. The mixture was then cooled and filtered using a funnel to separate the sulfonated carbon catalyst. After that, the filtrate was distilled using a rotary evaporator to obtain pure ethyl levulinate. The esterification results involved calculating the initial and final free fatty acid (FFA) content to determine the conversion of the esterification process. The FFA content (%) was determined using Eq. (2), VB is the volume of NaOH and *Mw* is the molecular weight of oleic acid (282.46 g mol^−1^) [29]. Then, the FFA conversion (%) is calculated using Eq. (3) where FFA_0_ is the initial FFA, and FFA_i_ is the final FFA. Thin layer chromatography (TLC) was used to confirm the esterification results. TLC was performed using silica gel 60 GF254 (Merck) and RP-18 F254s plates (Merck) with various solvent systems. In addition, the resulting product was also confirmed using gas chromatography-mass spectrometry (GC-MS, Agilent 7890A).

(2) $$\text{FFA}=\frac{\left(n_{B}\;.\;{\;V}_{B}\;.\;Mw\right)}{\left(w\;.\;1000\right)}\;x\;100\%$$

(3) $$\text{FFA}\;\text{Convertion}=\frac{{FFA}_{0}\;-\;{FFA}_{i}}{{FFA}_{0}}$$

## 3 Results and discussions

### 3.1 Moisture content and acid concentration of corncob carbon catalyst

The moisture content in corncob waste typically ranges from 80 to 90 % [30]. The weight loss of the sample after carbonization indicates the decomposition of compounds during the carbonization process. Table 2 shows data on water content and carbonization results from corncob waste. Corncob has a water content of 81.18 % with a carbon yield of 50.88 %. Next, the acid concentration in the catalyst is calculated using acid-base titration. This analysis focuses on the presence of acid groups (−SO_3_H, −COOH, and −OH) attached to the catalyst structure. The acid concentration in the catalyst is crucial as it acts as the active site for reactions and aids in the movement of the catalyst into the reaction mixture to minimize mass transfer resistance [31]. The acid concentration in the heterogeneous corncob catalyst is 1.26 mmol g^−1^ under the operating conditions of carbonization at 300 °C for 6 h and sulfonation for 8 h. In the research conducted by Tang, the acid concentration in the heterogeneous corncob catalyst is 1.55 mmol g^−1^ under the operating conditions of carbonization and sulfonation at 600 °C and 1 h [32]. The differences in acid concentration may occur due to variations in operating conditions in both the carbonization and sulfonation processes.

**Table 2:** Moisture content and carbonization process of corncob waste.

Moisture Content	Weight (g)	Percentage (%)
Corncob	1010 g	moisture content: 81.18 %
dried corncob	190 g	
Carbonization Process		
dried corncob	186.9 g	carbonization yield: 50.88 %
carbon yield	95.1 g	

### 3.2 Functional group analysis

**Fig. 1:**
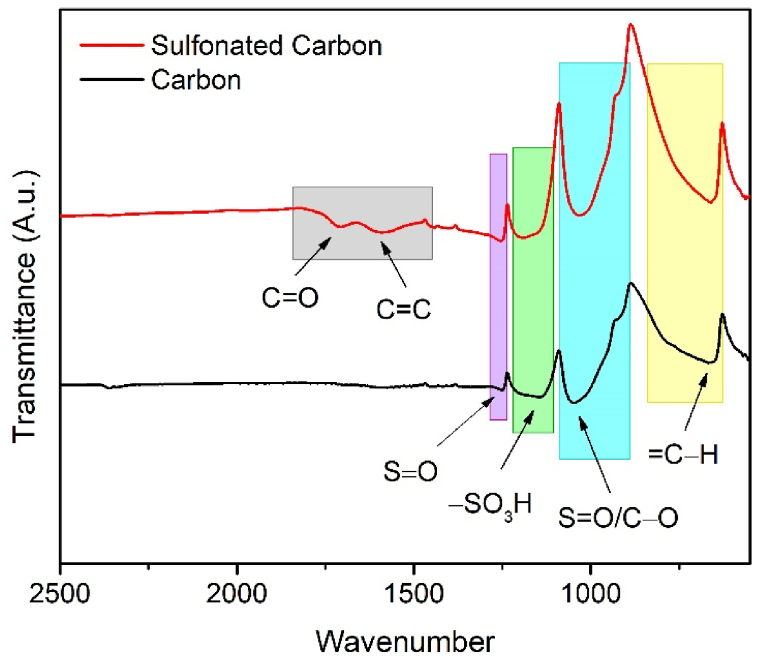
FTIR spectra of carbon and sulfonated carbon from corncob.

The catalyst was performed using FTIR to determine the presence of sulfonate groups attached to the carbon catalyst. Fig. 1 shows the FTIR spectra of the carbon and sulfonated carbon from corncob. Carbon samples show characteristic peaks of C−O groups (ether, hydroxyl, or ester) at 1020 cm^−1^ [33]. This peak overlaps with the symmetric vibration −SO_2_ which is at 1030−1100 cm^−1^ [34,35]. The peak at 1200-1100 cm^−1^ can be assigned to the asymmetric vibration absorption of the −SO_3_H group [21]. Apart from that, there is also a peak at 830–630 cm^−1^ which is the = C−H groups, which mostly consist of aldehyde compounds [36].

Meanwhile, in the sulfonated carbon, the intensity of the symmetric and asymmetric vibration peak of −SO_3_H appears to increase. This shows that the carbon already contains sulfonate groups, but when the sulfonation process is carried out, the number of sulfonate groups increases. These results are in accordance with previous research, where carbon from pyrolysis of corncob and cornsilk containing sulfur atoms which came from raw biomass materials [37]. In addition, the sulfonated carbon has an additional absorption band at 1713 cm^−1^ which indicates the presence of C=O groups (carbonyl, quinone, ester, or carboxyl), and at 1598 cm^−1^ which indicates C=C (alkenyl) groups at [38]. This peak can be caused by the C=C stretching mode of carbon caused by infrared activation in the sulfonation process [39]. This increase in sulfonate intensity and new representative absorption peaks indicate that the sulfonic acid groups were successfully grafted onto the catalyst surface.

### 3.3 SEM analysis

**Fig. 2:**
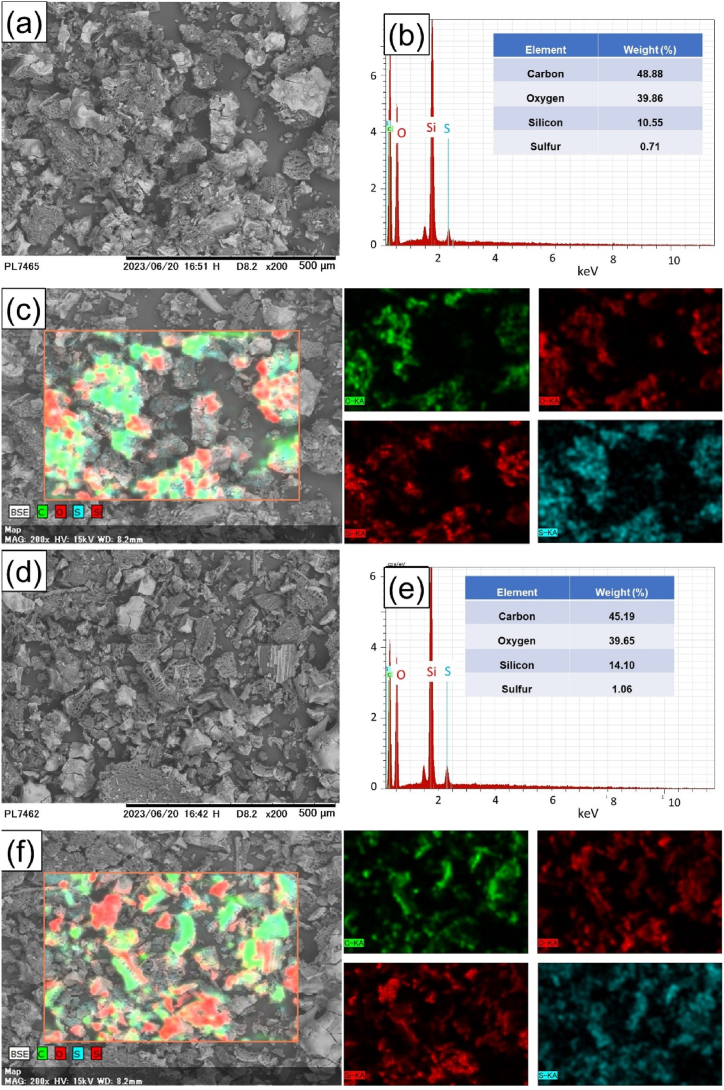
(a) SEM image, (b) EDS, and (c) mapping image of carbon, and (d) SEM image and (e) EDS, and (f) mapping image of sulfonated carbon.

The SEM-EDS analysis of the carbon catalyst from corncob can be observed in Fig. 2. The carbon and sulfonated carbon catalyst has an amorphous and irregular morphology, consistent with another research [40]. The size distribution of carbon particles is 50–150 μm with an average of 93 μm. In sulfonated carbon, the size decreases to 10−50 μm with an average of 30 μm. SEM-EDS analysis of the sulfonated carbon catalyst shows the detected carbon, oxygen, silicon, and sulfur elements [37]. Fig. 2a-c represents the SEM EDS analysis of carbon from corncob before sulfonation. Fig. 2d-f represents the SEM EDS analysis of carbon catalyst from corncob after sulfonation. There is a decrease in the carbon and an increase in the sulfur when comparing carbon before and after sulfonation. This indicates that the sulfonation process, involving the attachment of sulfonate groups to carbon, has been successfully carried out. The elemental mapping image shows that sulfur atoms are surrounding carbon and silicon atoms. This is in line with the FTIR results, which indicate the presence of sulfonate groups attached to the carbon catalyst from corncob. The presence of acid sites and acid strength on the catalyst can lead to higher esterification yields [5].

### 3.4 XRD analysis

**Fig. 3:**
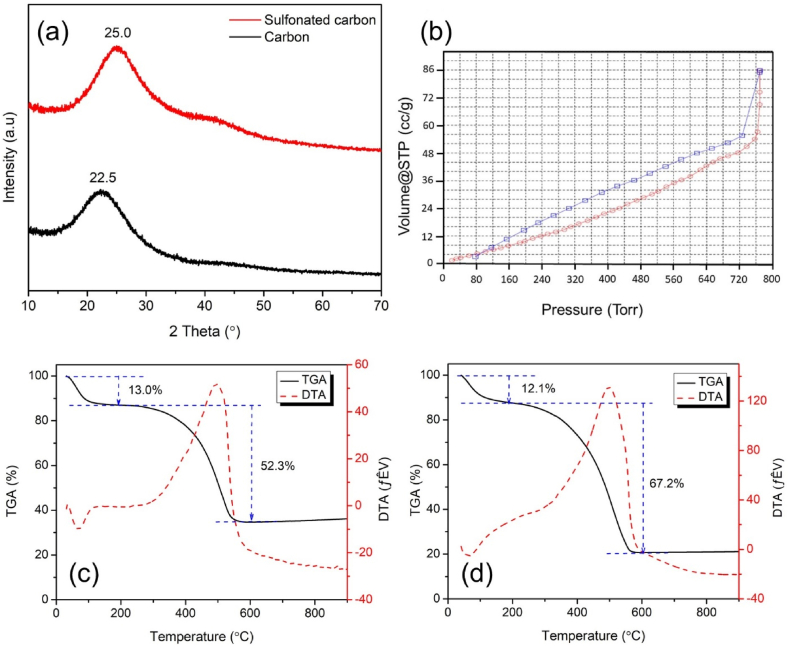
(a) XRD pattern, (b) N_2_ adsorption–desorption isotherms, and TG-DTA curve of (c) carbon and (d) sulfonated carbon catalyst from corncob.

The XRD pattern of the samples catalyst from corncob can be observed in Fig. 3a. The pattern shows that carbon have a broad peak at 2θ = 15–32° and sulfonated carbon 2θ = 17–34° is attributed to (002) planes of amorphous carbon structures containing randomly oriented aromatic carbon sheets [41]. The less intense and broader peak at 2θ = 38–48° corresponds to the (101) plane of the graphite structure [42]. There are shifts and different intensities between carbon before and after sulfonation. This is possibly due to the weakening of the carbon sheet due to the inclusion of sulfonate groups which increases the disruption of the carbon structure [5].

### 3.5 Nitrogen adsorption-desorption analysis

The specific surface area is important for the catalytic performance. Fig. 3b represents the BET plots of the sulfonated carbon sample. The specific surface area, BJH pore radius, and BJH pore volume were evaluated. The sulfonated carbon catalyst falls within the category of mesopores and exhibits a hysteresis type IV [43]. The sulfonated carbon has a specific surface area of 51.69 m^2^ g^−1^, a pore volume of 0.118 cm^3^ g^−1^, and a pore radius of 16.93 Å using the BJH method. A high specific surface area indicates a high physical adsorption capacity but does not necessarily have a direct relationship with chemical adsorption and catalytic reaction abilities [44].

### 3.6 TG-DT analysis

The TG-DTA curve shows the mass loss of the sample as a function of temperature. A decrease in temperature of 50–200 °C indicates the presence of adsorbed water in the sample. Meanwhile, a decrease at a temperature of 300–600 °C indicates carbon decomposition of various oxygen-containing groups (–COOH, –OH, –SO_3_H) [35]. The DSC diagram shows only an endothermic peak at 67 °C, related to the loss of adsorbed water, and an exothermic peak at 500 °C that is ascribed to carbon decomposition and elimination of oxygen-containing groups such as –SO_3_H [41]. The carbon sample has a water content of 13.0 %, with a carbon decomposition of 52.3 % (Fig. 3c). Meanwhile, sulfonated carbon has a water content of 12.1 %, with a carbon decomposition of 67.2 % (Fig. 3d). This shows that the sulfonated carbon sample has more oxygen-containing groups bound to the sample.

### 3.7 Catalysis activity using response surface methodology (RSM)

**Fig. 4:**
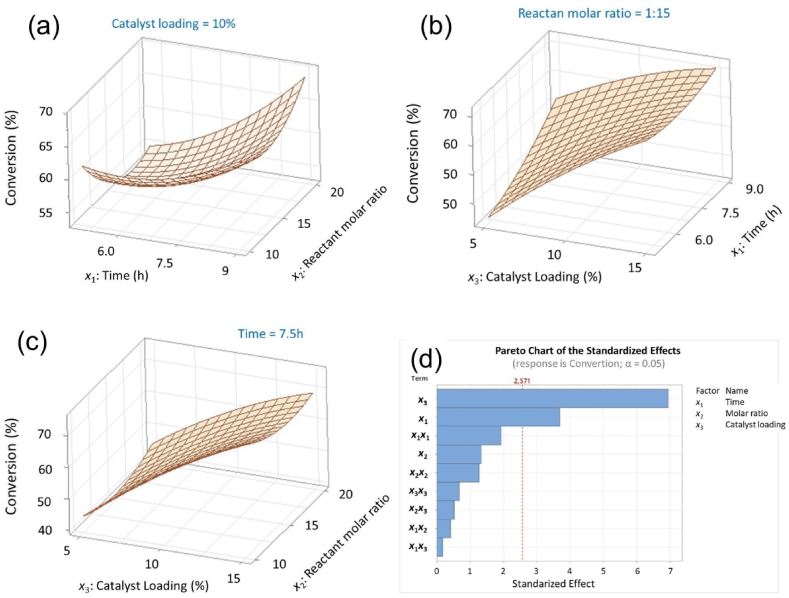
Surface plot for (a) time and molar ratio, (b) time and catalyst loading, (c) molar ratio and catalyst loading on levulinic acid conversion, and (d) pareto chart of statistical analysis results.

Fig. 4 shows the response surface of several parameters in the ethyl levulinate esterification process. The conversion of levulinic acid increases when the catalyst loading increases. The optimal catalyst loading that produces the highest levulinic acid conversion is 15 % (Fig. 4(a–c)). The increase in conversion due to catalyst loading is due to the increase in catalytically active sites on the catalyst [45]. However, excessive amounts of catalyst can reduce conversion. This is because the solution becomes thicker, potentially inhibiting the esterification reaction. Too much catalyst can hinder the mass transfer process. Increasing the viscosity of the mixture can inhibit catalyst activity [[46], [47], [48]]. For the effect of reaction time, the highest conversion was achieved at a reaction time of 9 h. The significant influence of time on the esterification reaction is indicated by the p-value in Fig. 4d, which is below 0.05. When varying the molar ratio of reactants, the conversion of levulinic acid increases at low ratios. The molar ratio of this reactant has a smaller influence on the conversion of levulinic acid produced. This is because excessive ethanol can reduce the concentration of levulinic acid, thereby reducing the efficiency of the esterification reaction. Excess ethanol in the mixture can cause the formation of side reactions, such as the production of ether compounds [49].

### 3.8 Reusability and kinetics studies of catalyst

**Fig. 5:**
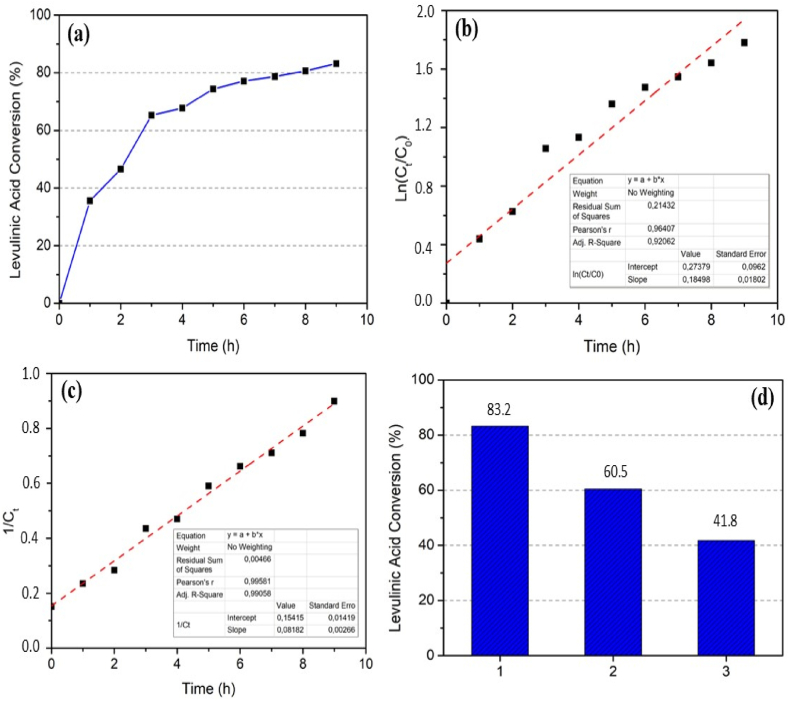
(a) Levulinic acid conversion, (b) first-order, (c) second-order kinetics reaction, and (d) catalytic reusability (Time = 9 h, catalyst loading = 10 %, reactant molar ratio = 1:10 (levulinic acid:ethanol).

The esterification reaction under operating conditions of 9 h, catalyst loading of 10 %, and a molar ratio of levulinic acid to ethanol of 1:10 resulted in the highest conversion of levulinic acid, namely 83.15 %. The enhancement of levulinic acid conversion and reaction kinetics are presented in Fig. 5a. The graph shows that after 9 h, the reaction is still ongoing, this shows that the reaction has not reached equilibrium. The kinetics study was simulated using first-order (Fig. 5b) and second-order (Fig. 5c) kinetics. The results show that the second order kinetics better fits the data obtained. The R-squared value of the second order is 0.9906 with a kinetic constant of 0.0818 L/mol.hour.

The catalytic reusability is performed to assess the performance of the catalyst. After use, sulfonate carbon is filtered, further washed with distilled water until it reaches a neutral pH, and then dried. Fig. 5d shows the catalyst reusability performance. The result shows that the catalyst can be reused for up to 3 cycles. The decrease in levulinic acid conversion from 83.2 to 41.8 % is attributed to the presence of reactants or products adhering to the catalyst's surface, which hinders the catalyst's proper functioning [50]. The decrease in levulinic acid conversion can be attributed to several factors, including contamination of the catalyst by O_2_, H_2_O, and CO_2_, structural changes occurring in previous experiments or during the regeneration process, and the potential degradation of active groups on the catalyst due to previous experiments or during the washing process [51]. These factors can all contribute to the reduced catalytic performance and the decline in conversion observed in subsequent reactions. To maintain catalyst activity and reusability, it is important to address these issues through appropriate regeneration and purification procedures.

## 4 Conclusions

Corncob can be utilized as a heterogeneous acid catalyst. After carbonization and sulfonation, the acid concentration in the heterogeneous catalyst is 1.26 mmol g⁻^1^. The sulfonated carbon exhibits increased peak intensities of symmetric and asymmetric vibrations of −SO_3_H indicating a higher amount of sulfonate groups than carbon before sulfonation. The sulfonated carbon catalyst has an amorphous and irregular morphology. There is a decrease in carbon and an increase in sulfur when comparing carbon before and after sulfonation, indicating the success of the sulfonation process. The sulfonated carbon at 2θ = 17–34° is a structure of amorphous carbon containing randomly oriented sheets of aromatic carbon. The sulfonated carbon catalyst falls into the mesoporous category and exhibits type IV hysteresis. The esterification results show the highest conversion under operating conditions of 9 h, 10 % catalyst loading, and a molar ratio of levulinic acid:ethanol = 1:10 which has 83.15 % conversion. The constant kinetics of the corncob catalyst reaction are 0.0818 L/mol.h. Catalyst reusability demonstrates that the corncob-derived carbon sulfonate catalyst can be used up to 3 times. This study indicates that corncob can be a potential raw material as a catalyst in the esterification of ethyl levulinate production.

## Data availability

Data will be made available on request.

## CRediT authorship contribution statement

**Dian Ratna Suminar:** Writing – original draft, Resources, Methodology, Data curation. **Cinantya Zahrina Pribadi:** Methodology, Formal analysis, Data curation. **Qonita Rahmah Fitriana:** Methodology, Data curation. **Eko Andrijanto:** Validation, Supervision. **Muhamad Diki Permana:** Visualization, Validation, Software, Methodology, Formal analysis, Data curation. **Diana Rakhmawaty Eddy:** Writing – review & editing, Validation, Supervision, Conceptualization. **Iman Rahayu:** Supervision, Funding acquisition.

## Declaration of competing interest

The authors declare that they have no known competing financial interests or personal relationships that could have appeared to influence the work reported in this paper.
